# Inactivation of the tight junction gene *CLDN11* by aberrant hypermethylation modulates tubulins polymerization and promotes cell migration in nasopharyngeal carcinoma

**DOI:** 10.1186/s13046-018-0754-y

**Published:** 2018-05-10

**Authors:** Hsin-Pai Li, Chen-Ching Peng, Chih-Ching Wu, Chien-Hsun Chen, Meng-Jhe Shih, Mei-Yuan Huang, Yi-Ru Lai, Yung-Li Chen, Ting-Wen Chen, Petrus Tang, Yu-Sun Chang, Kai-Ping Chang, Cheng-Lung Hsu

**Affiliations:** 1 Graduate Institute of Biomedical Sciences, Chang Gung University, No.259, Wenhua 1st Rd., Guishan Dist., Taoyuan City, 333 Taiwan; 2 Department of Microbiology and Immunology, Chang Gung University, No.259, Wenhua 1st Rd., Guishan Dist., Taoyuan City, 333 Taiwan; 3 Department of Medical Biotechnology and Laboratory Science, Chang Gung University, No.259, Wenhua 1st Rd., Guishan Dist., Taoyuan City, 333 Taiwan; 4 Department of Biomedical Sciences, Chang Gung University, No.259, Wenhua 1st Rd., Guishan Dist., Taoyuan City, 333 Taiwan; 5 Molecular Medicine Research Center, Chang Gung University, No.259, Wenhua 1st Rd., Guishan Dist., Taoyuan City, 333 Taiwan; 6 Bioinformatics Center, Medical School, Chang Gung University, No.259, Wenhua 1st Rd., Guishan Dist., Taoyuan City, 333 Taiwan; 7 Department of Otolaryngology-Head and Neck Surgery, Chang Gung Memorial Hospital, Chang Gung University, No.5, Fuxing St., Guishan Dist., Taoyuan City, 333 Taiwan; 8 Division of Hematology-Oncology, Chang Gung Memorial Hospital, Chang Gung University, No.5, Fuxing St., Guishan Dist., Taoyuan City, 333 Taiwan

**Keywords:** Nasopharyngeal carcinoma, Methylation, Tight junction, CLDN11

## Abstract

**Background:**

Aberrant hypermethylation of cellular genes is a common phenomenon to inactivate genes and promote tumorigenesis in nasopharyngeal carcinoma (NPC).

**Methods:**

Methyl binding domain (MBD)-ChIP sequencing of NPC cells, microarray data of NPC biopsies and gene ontology analysis were conducted to identify a potential tumor suppressor gene *CLDN11* that was both hypermethylated and downregulated in NPC. Bisulfite sequencing, qRT-PCR, immunohistochemistry staining of the NPC clinical samples and addition of methylation inhibitor, 5’azacytidine, in NPC cells were performed to verify the correlation between DNA hypermethylation and expression of *CLDN11*. Promoter reporter and EMSA assays were used to dissect the DNA region responsible for transcription activator binding and to confirm whether DNA methylation could affect activator’s binding, respectively. *CLDN11* was transiently overexpressed in NPC cells followed by cell proliferation, migration, invasion assays to characterize its biological roles. Co-immunoprecipitation experiments and proteomic approach were carried out to identify novel interacting protein(s) and the binding domain of CLDN11. Anti-tumor activity of the *CLDN11* was elucidated by in vitro functional assay.

**Results:**

A tight junction gene, *CLDN11*, was identified as differentially hypermethylated gene in NPC. High methylation percentage of *CLDN11* promoter in paired NPC clinical samples was correlated with low mRNA expression level. Immunohistochemistry staining of NPC paired samples tissue array demonstrated that CLDN11 protein expression was relatively low in NPC tumors. Transcription activator GATA1 bound to *CLDN11* promoter region − 62 to − 53 and its DNA binding activity was inhibited by DNA methylation. Re-expression of CLDN11 reduced cell migration and invasion abilities in NPC cells. By co-immunoprecipitation and liquid chromatography-tandem mass spectrometry LC-MS/MS, tubulin alpha-1b (TUBA1B) and beta-3 (TUBB3), were identified as the novel CLDN11-interacting proteins. CLDN11 interacted with these two tubulins through its intracellular loop and C-terminus. Furthermore, these domains were required for *CLDN11*-mediated cell migration inhibition. Treatment with a tubulin polymerization inhibitor, nocodazole, blocked NPC cell migration.

**Conclusions:**

*CLDN11* is a hypermethylated and downregulated gene in NPC. Through interacting with microtubules TUBA1B and TUBB3, CLDN11 blocks the polymerization of tubulins and cell migration activity. Thus, *CLDN11* functions as a potential tumor suppressor gene and silencing of *CLDN11* by DNA hypermethylation promotes NPC progression.

**Electronic supplementary material:**

The online version of this article (10.1186/s13046-018-0754-y) contains supplementary material, which is available to authorized users.

## Background

Aberrant DNA hypermethylation of tumor suppressor genes is a well-studied epigenetic gene silencing event, which promotes cancer formation and progression [1–3]. Nasopharyngeal carcinoma (NPC) is an Epstein–Barr virus (EBV)-associated head and neck cancer. The oncogenic viral latent membrane protein 1 (LMP1) can activate host DNA methyltransferase 1 (DNMT1), causing aberrant hypermethylation, transcription inactivation, and loss of function of tumor suppressor genes in NPC [4–8]. We previously demonstrated that HoxA2 (coding gene) [9] and miR-148a (noncoding gene) [10] are the two tumor suppressor genes silenced through aberrant DNA hypermethylation in NPC tumors.

**Fig. 1:**
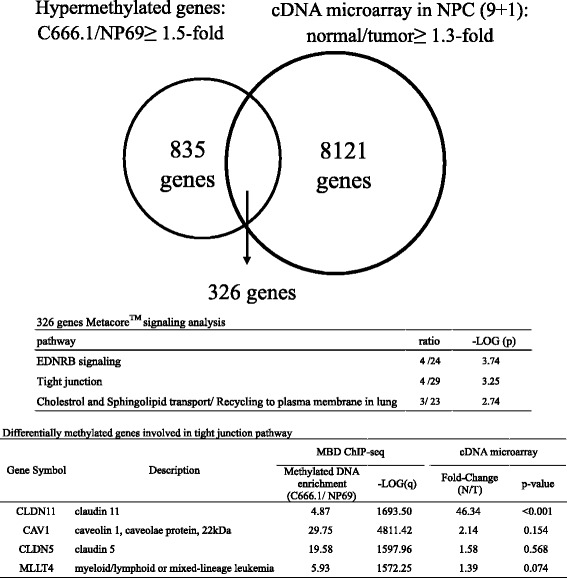
Screening for potential hypermethylated genes in NPC. The Venn diagram indicates intersected 326 genes that are both hypermethylated in NPC cells with relative methylated DNA enrichment ≥1.5-fold in C666.1 compared with that of NP69 (1161 genes) and downregulated at least 1.3-fold in nine NPC tumors (T) compared with pooled adjacent normal tissues (N) (8447 genes). The intersected genes were analyzed by MetaCore™ GeneGo pathway analysis. The top three significant pathways are listed. The bottom table shows the relative methylated DNA enrichment and the expression fold-change of the four genes involved in the tight junction pathway

To characterize genome-wide, aberrantly hypermethylated and downregulated genes in NPC cells, we employed next generation sequencing (NGS) approach to identify the NPC-specific methylated genes. First, we enriched the methylated genomic DNA from NPC cell line C666.1 (sample) and immortalized normal NP cell line NP69 (control), respectively, by using methylated DNA affinity column containing methyl binding protein, MBD (Methylminer Methylated DNA Enrichment Kit). We then collected and PCR-amplified the methylated DNA from the methylated DNA affinity column. The amplified DNA was subjected to high-throughput NGS sequencer (Illumina GAII; Genomic Center Yang Ming University, Taiwan). Sequencing data were further analyzed by DNAnexus ChIP-seq algorithm. Significantly enriched and differentially methylated genes (experimental versus background ≥1.5) in C666.1 when compared with that of the NP69 were identified (Fig. 1). These NPC-specific methylated genes are potential tumor suppressor genes which could be epigenetically inactivated during NPC tumorigenesis. Second, we intersected the above NPC hypermethylated gene list (NGS) with the downregulated genes selected from our in-house NPC tumors cDNA microarray (Affymetrix HG U133 plus 2.0 chip) to correlate the notion that hyperemethylated genes are often transcriptionally repressed. The cDNA expression profiling was compared between nine NPC tumor samples and one pooled adjacent normal tissues [11]. Gene expression > 1.3-fold reduction in NPC tumor tissues when compared with that of pooled non-tumors were selected. After intersecting the two gene lists, we identified a differentially methylated and downregulated gene candidate *claudin 11 (CLDN11)* in NPC cells. Claudins are a family of genes with 27 members. They are integral membrane proteins containing four transmembrane domains which serve as crucial tight junction components and cell barrier for cells [12–17]. *CLDN11* is hypermethylated and silenced in bladder cancer [18], gastric cancer [19], oral leukoplakias [20] and malignant melanoma [21]. The reduction in *CLDN11* expression is associated with increase in invasiveness in multiple cancer types [18, 22, 23]; the reintroduction of this gene reverses the cancerous phenotype, suggesting that *CLDN11* has a tumor suppressive role. However, the underlying mechanism remains unclear.

In this study, we observed that the methylation percentage of the *CLDN11* promoter inversely correlated with the CLDN11 expression in NPC tumors. Aberrant DNA methylation of the *CLDN11* promoter prevents the binding of the transcription activator GATA1 near the transcription start site, resulting in gene silencing. We also dissected CLDN11 protein domains responsible for the inhibition of cell migration function. Two cellular tubulins TUBA1B and TUBB3 were identified to be the novel proteins interacting with CLDN11. The interaction between CLDN11 and these tubulins is necessary for the CLDN11-mediated cell migration inhibition in NPC cells. Tubulins are the basic subunits of microtubules of which form structural network of cytoskeleton in cytoplasm. It has been known that polymerization and depolymerization of tubulins are essential for cellular processes such as cell movement, transportation of vesicles and organelles, centrosome assembly, and segregation of chromosomes during mitosis and meiosis. Cellular proteins interacting with microtubules are known as microtubule associated proteins (MAPs) [24–26]. The biological significance of the interaction between CLDN11 and tubulins will be further discussed.

## Methods

### Cell lines, NPC tissue samples

The NPC cell lines (C666.1 and HK1) and immortalized normal NP cells (NP69) were gifts from Dr. S. W. Tsao (Hong Kong University, SAR, China). NPC cells were cultured in 10% fetal bovine serum (FBS) (Thermo Fisher Scientific/Gibco, Waltham, MA)/RPMI1640; NP69 cells were maintained in defined keratinocyte-SFM with growth supplement (Thermo Fisher/ Gibco10744019). The TW02 and TW06 cells were established by Dr. C. T. Lin (National Taiwan University, Taiwan) and cultured in 10% FBS/DMEM (Thermo Fisher/ Gibco). NPC cells were treated with 10 μM demethylation agent 5’Aza for 5 days, with daily replacement of fresh medium. Seven pairs of frozen NPC tumor and adjacent normal biopsies (< 2 mm) were collected from Chang Gung Memorial Hospital by Dr. K. P. Chang. Genomic DNA and RNA of NPC biopsies were simultaneously extracted by using the TRIZOL reagent (Thermo Fisher/Invitrogen) (Additional file 1).

### DNA transfection

TW02 (4 × 10^5^) or HK1 (8 × 10^5^) cells were seeded in 6-well plate ~ 16 h prior to DNA transfection. Cells were 60~ 80% confluent at the time of transfection. DNA (2 μg) and transfection reagent (4 μl) jetPRIME (Polyplus, France) were diluted in jetPRIME buffer (200 μl). The mixture was incubated at room temperature for 10 min and was added dropwise into the medium. Transfection medium was replaced after 5 h and cells were harvested 48 h post transfection.

### Construction of luciferase reporters and expression vectors

Genomic DNA from NP69 was used as template for the amplification of the promoter regions of *CLDN11* (− 900~ + 197; − 477~ + 197; − 213~ + 197 and − 10~ + 197). PCR products were then subcloned into *Bgl*II and *Hind*III restriction enzyme sites of pGL3Basic (Promega, Madison, WI). Mutant primers were used to generate the GATA sites directed mutants (− 92/HindIII; − 62/HindIII; and + 184/KpnI) of *CLDN11* promoter luciferase reporters. RNA from normal human peripheral blood cells was reverse transcribed to cDNA. The full length and deletion CLDN11 cDNA clones were PCR-amplified and subcloned into the *Bam*HI and *Hind*III sites of the pCMV-3tag-8 vector (Stratagene, La Jolla, CA) to generate different Flag-tagged clones.

### Bisulfite sequencing

Genomic DNA from NPC patients and cell lines (C666.1, HK1, NP69) with or without 5’Aza treatment were purified by using Genomic DNA mini Kit (Thermo Fisher/Invitrogen) and 1.8 μg DNA was treated with sodium bisulfite by using a Zymo DNA Modication Kit (Zymo Research, Hornby, Canada) and amplified by specific bisulfite sequencing (BS) primers that were designed by using Methprimer (http://www.urogene.org/methprimer/, UCSF) [27] and listed in Additional file 2: Table S1. The amplified PCR products were ligated into yT&A vector (Yeastern Biotech, Taiwan). Eight individual clones were sequenced to determine promoter methylation status.

### RNA extraction and quantitative real-time PCR

Total RNA was extracted and DNaseI treated. Reverse transcription (RT) was performed by using Improm-II reverse transcriptase (Promega) according to the manufacturer’s protocol. Q-RT-PCR was performed by using SYBR master mixture (Kapa Biosystems, Woburn, MA) on an IQ5 system (Bio-Rad, Hercules, CA), according to the manufacturer’s instructions. The relative gene expression level was determined with respect to that of the β-actin*,* and calculated by the 2^-∆Ct^ method.

### Promoter analysis and luciferase assay

To normalize each transfection reaction, a renilla reporter plasmid (20 ng pRL-CMV; Promega) was co-transfected with the luciferase reporters (2 μg). Cells were harvested 48 h post-transfection and lysed, and promoter activity was assayed by using the Dual Luciferase Assay System (Promega) on a GLOMAX 20/20 Luminometer (Promega).

### DNA pull-down assay

Double-stranded biotinylated probes (100 pmol) were incubated with 50 μl M-280 Streptavidin Dynabeads (Thermo Fisher/Invitrogen) in 5 mM Tris-HCl pH 7.5, 0.5 mM EDTA, 1 M NaCl for 1 h. Nuclear extracts overexpressing flag-tagged GATA1–3F and GATA2-3F (100 μg) were precleared by binding to Streptavidin Dynabead (25 μl) followed by incubation with the probe-bounded beads. The Dynabead-bound complexes were washed six times with binding buffer containing 0.5% NP40. DNA-bound proteins were eluted in SDS sample buffer and verified by Western blotting.

### Electrophoretic mobility shift assay (EMSA)

EMSA was performed as described previously [9].

### Cell proliferation, migration and invasion assay

Transient transfected CLDN11 and vector control of TW02 (5 × 10^4^) or HK1 (2 × 10^5^) cells were seeded in 6-well plates, and cell numbers were counted daily for 5 days. Cell proliferation was determined by trypan blue (Thermo Fisher/Gibco) exclusion and cell counting. Cell migration and invasion were performed by using the 8 μm pore size BD Falcon 24-well insert system and BD Matrigel BioCoat (Corning, NY), respectively. For migration assays, TW02 cells (4 × 10^5^) or HK1 (8 × 10^5^) in 0.5 ml serum free medium were seeded to the upper chamber with the lower chamber containing 10% FBS/medium (0.8 ml) and cultured for 24 h, allowing cells to migrate through the 8 μm membrane. TW02 or HK1 cells were treated with or without tubulin polymerization inhibitor nocodazole (Sigma). For invasion assays, TW02 cells (4 × 10^5^) or HK1 (8 × 10^5^) were seeded to the upper chamber coated with Matrigel with the lower chamber containing 10% FBS/medium and cultured for 24 h (TW02) or 72 h (HK1). The inserts were stained with 10 mg/ml crystal violet in 70% ethanol and 2% formaldehyde and destained with tap water. The membrane was then excised and stained cells from 10 microscopic fields were counted. All experiments were repeated at least three times.

### Antibodies and recombinant proteins

Primary antibodies: Flag antibodies (M2, Sigma and MDBio, Taiwan), Actin monoclonal antibody (Millipore, Billerica, MA, USA), GATA1 antibody (MDBio), CLDN11 antibodies (Western/ab53041, Abcam, Cambridge, UK; IHC/SC25711, Santa Cruz, Dallas, TX), Tubulin alpha IB (ab108629, Abcam) and Tubulin beta III antibodies (ab52623, Abcam). Secondary antibodies: HRP-conjugated goat anti-rabbit and goat anti-mouse antibodies (Gene Teks) were used. Western blot results were visualized by ECL detection kit (Immobilon, Merck/Millipore, Germany). Recombinant proteins GST-GATA1 (H00002623, Abnova, Taiwan) and GST-CLDN11 (H00005010, Abnova) were used in EMSA and tubulin polymerization assay, respectively.

### Immunofluorescence and immunohistochemical staining

TW02 cells (1X10^5^) were grown on polylysine-coated coverslip for 24 h and followed by transfection of pCMV/CLDN11–3F or vector control plasmids. Post-transfection 24 h, cells were fixed with 4% formaldehyde, permeabilized with 0.1% Triton X-100/PBS and blocked with 5% BSA/PBS. The coverslips were incubated with the primary antibodies for 2 h at room temperature, followed by incubation with appropriate FITC- or rhodamine-conjugated secondary antibodies (Jackson ImmunoReasearch, West Grove, PA). Finally, the nuclei were stained with DAPI fluorescent dye (AAT Bioquest, Sunnyvale, CA). Coverslips were mounted with the VECTASHEILD reagent (Vector Laboratories, CA). The images were acquired by confocal microscopy (Zeiss, LSM780), CGU, Microscope Center. Immunohistochemistry was performed by CGU, Pathology Core Laboratory by using NPC tissue array (NPC961; Pantomics, Richmond, CA).

### Statistical analysis

**Fig. 2:**
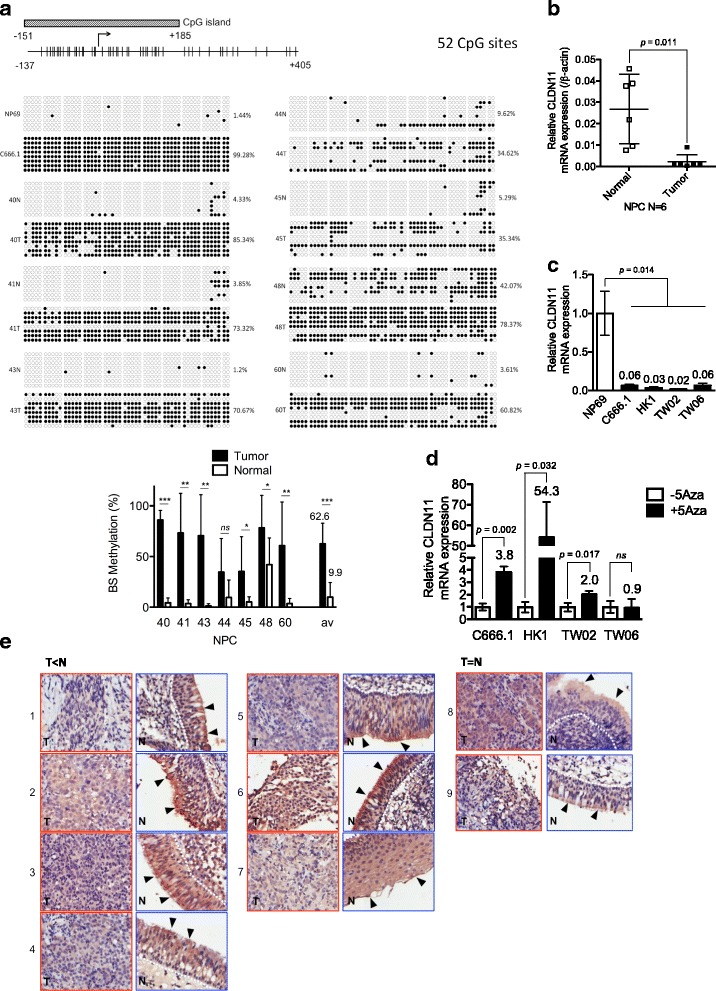
Identification of the hypermethylated and downregulated gene *CLDN11* in NPC. **a** Bisulfite sequencing analysis was performed on − 137 to + 405 in C666.1 and NP69 cells, and seven paired NPC clinical samples. Each horizontal row represents a single clone; the methylation percentages of at least eight individual clones are indicated as unmethylated (○) and methylated (●) CpG sites. The lower panel shows the average methylation percentage for each sample. qRT-PCR analysis of CLDN11 mRNA expression was performed in (**b**) 6 paired NPC tissues and (**c**) NP69 cells and four NPC cell lines. The results were normalized to β-Actin expression. **d** Columns represent the relative fold-change of the restored CLDN11 mRNA expression normalized with respect to β-Actin expression in NPC cell lines with (+) or without (−) 10 μM 5’Aza treatment. **e** Immunohistochemistry staining analysis of CLDN11 protein expression in nine paired NPC tissue arrays (Pantomics). Tumor tissues (T) and the corresponding adjacent normal tissues (N) are indicated. The results are shown at 200× magnification (CLDN11 staining intensity T < N: 7 pairs, T = N: 2 pairs). Higher magnification is shown in Additional file 4: Figure S2. White dotted lines mark the border of basement membrane of normal epithelial cells; black triangles indicate the apical membranous staining signals of CLDN11

**Fig. 3:**
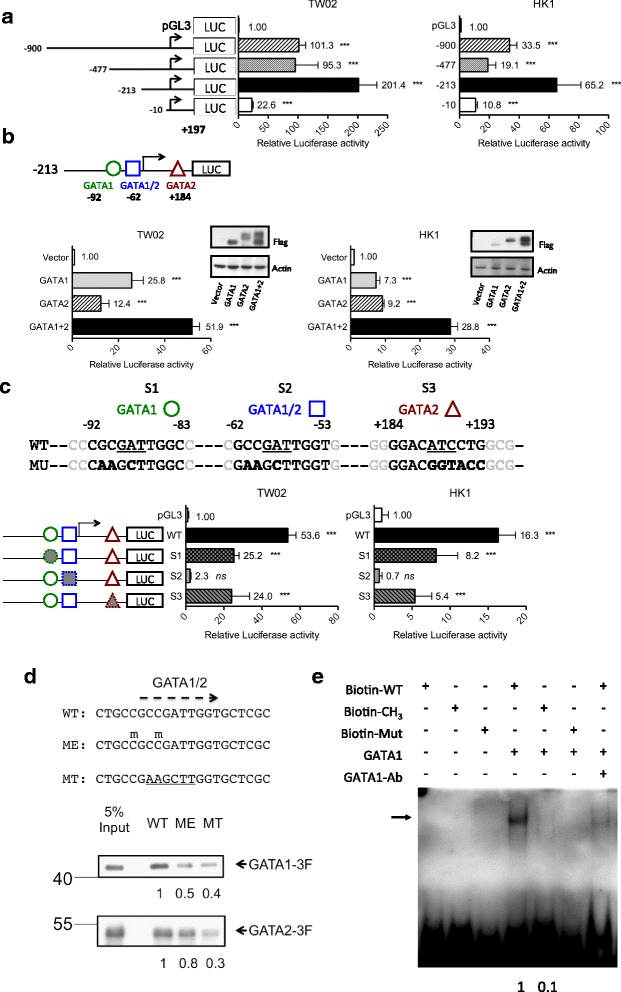
Methylation inhibits *CLDN11* promoter activity by impairing the binding of the transcriptional activator GATA1. **a** Different promoter activities of a series of *CLDN11* promoter deletion luciferase reporters were assayed in TW02 and HK1 cells. Intergroup comparison was conducted relative to the empty vector (pGL3). **b** The minimal *CLDN11* promoter reporter (− 213 to + 197) and the FLAG-tagged GATA1 and GATA2 expression clones were cotransfected into TW02 and HK1 cells. Three putative GATA binding sites: GATA1 (− 92), GATA1/2 (− 62), and GATA2 (+ 184), are indicated. GATA1: GATA1 overexpression, GATA2: GATA2 overexpression, GATA1 + 2: GATA1 and GATA2 co-overexpression. Intergroup comparison was conducted relative to the vector control. The expression levels of ectopic GATA-1, GATA-2, and actin (internal control) were examined through Western blotting. **c** Minimal *CLDN11* promoter reporters with wild-type (WT) and three mutated GATA binding sites (MU, S1–S3) were transfected into TW02 and HK1 cells, respectively. Intergroup comparison was conducted relative to the empty vector (pGL3). **a**~**c** All the luciferase reporter activities were normalized with respect to renilla activity. **d** DNA pull-down assay was used to analyze the binding affinity of exogenous FLAG-tagged GATAs (GATA1–3F and GATA2-3F) to WT, methylated (ME), and mutated (MT) biotinylated probes containing GATA1/2 site; the sequences of these probes are shown in the upper panel. Methylated cytosine is indicated by using “m” above C, and mutated sequences are underlined. Anti-GATA1 and anti-FLAG antibodies were used for examining the amount of bound GATA1–3F and GATA2-3F in the immunoprecipitates and 5% input. **e** EMSA was performed to compare the binding affinity of purified recombinant GATA1 for WT, ME, and MT biotinylated probes of the GATA1/2 site. The arrow indicates DNA–protein complexes. Antibodies against GATA1 were used for supershift assays

**Fig. 4:**
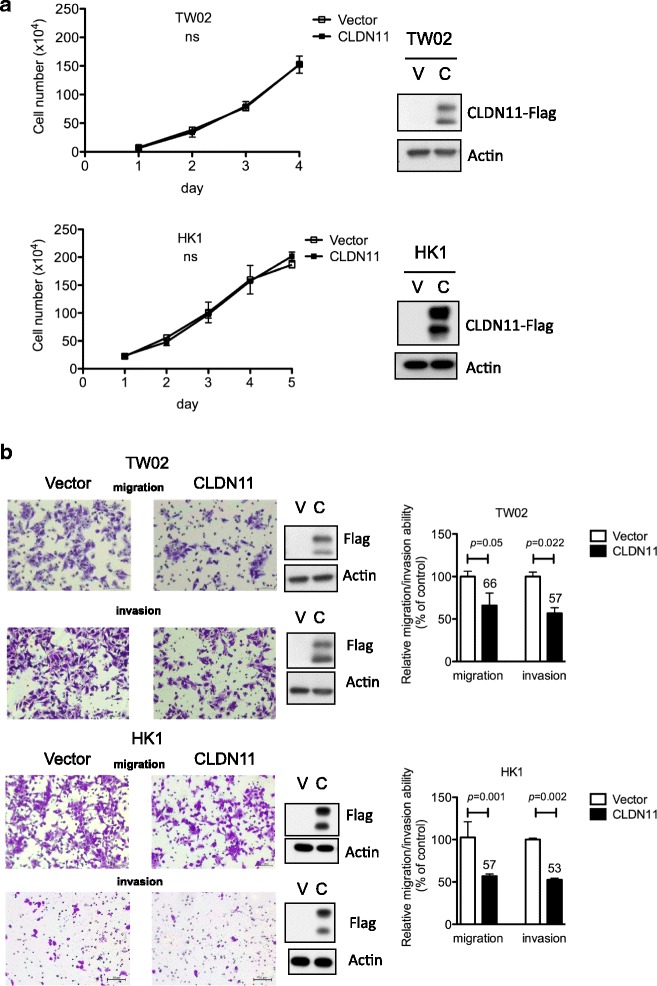
*CLDN11* reduces migration and invasion abilities of NPC cells. **a** Cell number (proliferation) was monitored in TW02 and HK1 cells expressing CLDN11 (C) or vector control (V) for 5 days. **b** Transwell cell migration and invasion were assayed in TW02 and HK1 cells expressing CLDN11 (C) or vector control (V). Migrated or invaded cells were counted in 10 microscopic fields. The data are presented as percentage of cell migration or invasion relative to control. The expression of FLAG-tagged CLDN11 and actin (internal control) was detected by using specific antibodies (anti-Flag and anti-actin)

**Fig. 5:**
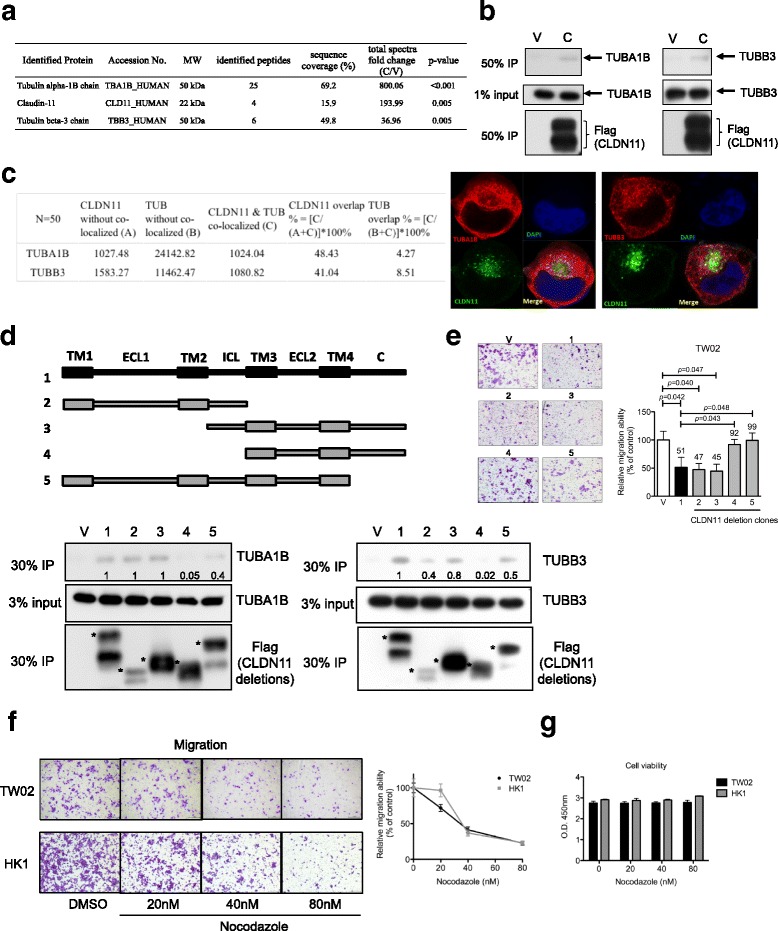
TUBA1B and TUBB3 are the interacting proteins of CLDN11; CLDN11 blocks cell migration by interfering with tubulin polymerization. **a** TW02 cells transfected with FLAG-tagged CLDN11 construct (C) or vector control plasmid (V) were harvested after 48 h. TW02 cell extracts and M2 beads were used for a co-immunoprecipitation assay. CLDN11-interacting proteins identified through LC–MS/MS and top-ranking proteins are listed in the table. **b** Immunoblot analyses were performed to confirm the interaction between FLAG-tagged CLDN11 and endogenous TUBA1B (left panel) or TUBB3 (right panel). **c** Subcellular distribution of exogenous FLAG-tagged CLDN11 and endogenous TUBA1B or TUBB3 in TW02 cells was assayed through immunofluorescence staining 24 h after transfection. The fluorescent signal (pixel) of each individual cell was quantitated by using the Zen 2.0 software (*n* = 50). The proportion of colocalized fluorescent signals (%) is indicated (FLAG-tagged CLDN11, green; Tubulins, red; DAPI, blue). **d** Schematic illustrations of WT and four deletion FLAG-tagged CLDN11 molecules—transmembrane (TM), extracellular loop (ECL), intracellular loop (ICL), and C-terminus (C). Various FLAG-tagged CLDN11 deletion clones or a vector control were used to dissect the interacting domains on CLDN11 that are crucial for the interaction of endogenous TUBA1B and TUBB3. Input (3%) and immunoprecipitates (30% IP) were assayed through immunoblot analysis by using appropriate antibodies (anti-Flag, anti-TUBA1B and anti-TUBB3). The asterisks denote the major bands of ectopic CLDN11 in the immunoblot assays. **e** CLDN11 deletion clones were used to perform cell migration assay and to determine which domains on CLDN11 are necessary for blocking cell migration in TW02 cells. **f** Cell migration and (**g**) cell viability assays were performed in the presence or absence of nocodazole in TW02 or HK1 cells

Values on bar graphs and curves were displayed as mean ± standard deviation (SD). For MBD ChIP-seq analysis, data were expressed as fold-enrichment of normalized experiment tags over normalized background tags within regions and compared by false discovery rate adjusted *p* value (q value) and analyzed by the DNAnexus platform (DNAnexus, CA, USA). For NPC microarray analysis, gene expression in 9 NPC tumor was compared with that of “one-combined non-tumors” by using the one-way analysis of variance (ANOVA) by Partek Genomics Suite (Partek, MO, USA). Intergroup comparisons were conducted by using the unpaired two-sample *t* test (Fig. 2, Fig. 3, Fig. 4, Fig. 5) or paired two-sample *t* test (Fig. 2b). A *p*-value less than 0.05 (*), 0.01 (**), 0.001 (***) was considered statistically significant. All graphed plots and statistical analyses (Fig. 2, Fig. 3, Fig. 4, Fig. 5) were conducted by the Prism 5 software (GraphPad) .

## Results

### Tight junction *CLND11* is a potential hypermethylated and downregulated gene in NPC

Two gene lists (1) differentially methylated genes (1161) and (2) downregulated genes (8447) were intersected. There were 326 gene candidates identified as potential genes inactivated because of aberrant DNA methylation (Fig. 1). To further understand the contribution of the inactivation of these genes toward NPC carcinogenesis, we further analyzed them by using gene ontology pathway analysis software MetaCore (Thomson Reuters, Toronto, Canada). The tight junction pathway is a significant pathway involved in cell adhesion (−log *P* = 3.25; Fig. 1). The tight junction-related gene *CLDN11* was selected because the relative *CLDN11* methylated DNA enrichment was high (4.87-fold) in NPC cells (Fig. 1 and Additional file 3: Figure S1) and the CLDN11 mRNA expression reduction fold-change in NPC tissues was also high (46.34-fold, Normal/Tumor; Fig. 1), suggesting that *CLDN11* is hypermethylated and downregulated in NPC.

### *CLDN11* methylation status is conversely correlated with its expression in NPC

To investigate the differentially methylated CpG sites of the *CLDN11* promoter in clinical samples, we performed bisulfite sequencing analysis of seven paired tumor and nontumor genomic DNA ranging from − 137 to + 405 containing 52 CpG sites. The results showed that *CLDN11* promoter was hypermethylated in seven NPC tumor tissues (34.62%–85.34%, average 62.64%) compared with that of the paired adjacent normal tissues (1.20%–42.07%, average 10%; Fig. 2a). The methylation percentage of the same promoter region was also higher in the NPC cells C666.1 (99.28%) than that of immortalized normal NP cells NP69 (1.44%), indicating that *CLDN11* is hypermethylated in NPC tumors and cells.

To correlate the methylation status with the mRNA expression level, we performed qRT-PCR to examine CLDN11 mRNA expression levels in the same NPC tissues and in various NPC cells. The data demonstrated that CLDN11 mRNA expression was significantly reduced (4.3–53.5-fold, average 22.5-fold) in NPC tissues versus adjacent normal tissues (Fig. 2b, *P* = 0.004). Similarly, CLDN11 mRNA expression levels decreased 15–50-fold in NPC cells (C666.1, HK1, TW02 and TW06) compared with those in NP69 cells (Fig. 2c). Furthermore, CLDN11 mRNA expression levels were restored upon DNA methylation inhibitor, 5’Aza, treatment in the three NPC cells C666.1, HK1, and TW02, but not in TW06 (Fig. 2d), suggesting that transcriptional repression of *CLDN11* by DNA methylation is occurred in NPC cells.

We next performed immunohistochemical staining by using NPC tissue array (Pantomics, Richmond, CA) to examine the CLDN11 protein expression levels in NPC. Seven of nine NPC tumors had lower CLDN11 expression levels (Fig. 2e, T<N #1–7 and Additional file 4: Figure S2) compared with their normal counterparts; by contrast, two NPC tumors (Fig. 2e, T=N #8–9 and Additional file 4: Figure S2) had similar CLDN11 expression compared with the adjacent normal tissues. In addition to the reduction in CLDN11 expression, NPC tumor cells were morphologically distinct from the normal epithelial cells, becoming disorganized and having irregular cell boundary with large nuclei. A paired normal tissue (#7 N) already demonstrated hyperplasia condition (presence of ≥4 cell layers above the basement membrane); nevertheless, CLDN11 expression remained relatively higher compared with its tumor tissue. Thus, our data supported that *CLDN11* methylation status is conversely correlated with mRNA expression and that DNA hypermethylation is responsible for the reduction in CLDN11 mRNA and protein expression levels in NPC.

### DNA methylation causes *CLDN11* inactivation by interfering with GATA binding toward the *CLDN11* promoter

To study the underlying molecular mechanism by which methylation inactivates *CLDN11* transcription, a series of deletion *CLDN11* promoter luciferase reporters were constructed and the transcriptional regulation of *CLDN11* and the key transcription factors involved were clarified (Additional file 5: Figure S3). Luciferase assay results indicated that in TW02 and HK1 cells, the minimal *CLDN11* promoter sequences are located within − 213 to + 197, *pCLDN11*(− 213) (Fig. 3a). We then predicted transcription factor binding sites within the *CLDN11* minimal promoter on the basis of the TFSEARCH website and selected transcription factor binding consensus, which overlapped with previous differentially methylated CpG sites. Consequently, GATA1 and GATA2 were the prominent transcription regulators for *pCLDN11*. To investigate whether GATA1 or GATA2 transactivates *CLDN11* promoter activity, each GATA expression plasmid and *pCLDN11*(− 213) were cotransfected into TW02 and HK1 cells, separately. Elevated *pCLDN11*(− 213) promoter activities were detected after ectopically expressing GATA1 or GATA2 (Fig. 3b), suggesting that both GATA1 and GATA2 act as transcriptional activators for the *CLDN11* promoter. Three putative GATA-binding motifs are present within the minimal *CLDN11* promoter: GATA1 site (− 92 to − 83), GATA1 and 2 site (− 62 to − 53), and GATA2 site (+ 184 to + 193) (Fig. 3c). To characterize which site is responsible for *CLDN11* promoter activation, we mutated each GATA consensus sequences as shown in Fig. 3c (upper panel; S1, GATA1 site: CGCGATTGGC to CAAGCTTGGC; S2, GATA1/2 site: GCCGATTGGT to GAAGCTTGGT; S3, GATA2 site: GGACATCCTG to GGACGGTACC). The activity of the GATA-mutated promoters decreased to ~ 50% in S1 and S3 and significantly reduced to 5% in S2 compared with that of the wild type *pCLDN11*(− 213) in both TW02 and HK1 cells (Fig. 3c), indicating that GATA1 and GATA2 activate the *CLDN11* promoter mainly through S2, a GATA1/2 site situated at a close proximity to the transcription start site.

To determine whether the binding of GATA1 and GATA2 toward the GATA1/2 site is interfered by DNA methylation, we performed DNA pull-down assays by using wild type (WT), methylated (ME), and mutated (MT) biotinylated probes containing GATA1/2 site (− 67 to − 47) and nuclear extracts from TW02 cells overexpressing either FLAG-tagged GATA1–3F or GATA2-3F. Both DNA methylation and mutation of GATA consensus sequences reduced the binding of either GATA1–3F or GATA2-3F when compared with that of the WT probes (Fig. 3d). However, the degree of inhibition of GATA1–3F binding is slightly greater than that of GATA2-3F toward the same methylated probe. Similarly, we used the same probes to bind purified recombinant GST-GATA1 and performed electrophoretic mobility shift assay (EMSA) experiments. Consequently, the recombinant GATA1 bound well to the WT probe but poorly to the methylated probe; less than ~ 10% of the binding was detected in the methylated probe (Fig. 3e). These data indicated that methylation impairs the binding of transcription activator GATA1 and leads to transcriptional inactivation of *CLDN11* in NPC.

### CLDN11 inhibits cell migration and invasion in NPC cells

Silencing of TSGs through promoter hypermethylation is an early event during cancer formation [2]. To characterize the biological roles of *CLDN11* in NPC, we performed cell proliferation, migration, and invasion assays in TW02 and HK1 cells transiently overexpressed with FLAG-tagged CLDN11–3F or the vector control. CLDN11 did not affect the cell proliferation ability (Fig. 4a) but significantly reduced cell migration (~ 40%) and invasion (~ 50%) abilities compared with the vector control in NPC cells (Fig. 4b), demonstrating that CLDN11 inhibits cell migration and invasion in NPC cells.

### Intracellular loop and C-terminus of CLDN11 are required for TUBA1B and TUBB3 interaction and CLDN11-mediated anti-migration

To understand the mechanism of CLDN11-mediated migration suppression, we identified the interacting proteins of CLDN11. We performed co-immunoprecipitation assays of FLAG-tagged CLDN11 (C) and vector control (V) by using FLAG M2 beads. The precipitated proteins were resolved on SDS-PAGE and stained by using Coomassie blue (Additional file 6: Figure S4). Each lane from the gel was sliced, trypsin-digested, and analyzed through liquid chromatography–tandem MS (LC–MS/MS) (CGU Proteomics Center). The resulting MS and MS/MS spectra were compared with the Human v3.26 database by using the Mascot algorithm. The spectral counting label-free quantification method [28] was used to quantitate the protein samples. Putative CLDN11 interacting proteins (Additional file 7: Table S2) were selected on the basis of the ratio of total spectra numbers of fold-change (> 30) enriched in CLDN11 versus control (*P* < 0.01). Significant interacting proteins tubulin alpha-1B (TUBA1B) and tubulin beta-3 (TUBB3) as well as the bait CLDN11 itself were identified; their corresponding spectra numbers of fold-change (C/V) are listed in Fig. 5a (peptide sequences detected are listed in Additional file 8). We subsequently performed co-immunoprecipitation and Western blotting assays to validate whether CLDN11 can interact with endogenous TUBA1B and TUBB3. FLAG-tagged CLDN11–3F and vector control were transiently overexpressed in NPC cells individually and used to immunoprecipitate endogenous TUBA1B and TUBB3, respectively. Our data indicated that exogenous CLDN11–3F indeed associated with small amount of cytoskeleton proteins TUBA1B and TUBB3 (Fig. 5b).

We next overexpressed FLAG-tagged CLDN11–3F in TW02 cells and observed the subcellular localization and distribution of the exogenous CLDN11–3F and endogenous cytoskeleton proteins TUBA1B and TUBB3 through confocal microscopy. The fluorescent signal of these proteins was quantitated and analyzed (Fig. 5c). Although the exogenous CLDN11–3F was overexpressed, the measured signal of CLDN11–3F was approximately 10 times lower than that of the endogenous tubulin, reflecting that the difference in the expression levels of CLDN11–3F and tubulins was rather high. Nearly half of the exogenous CLDN11–3F (41%–48%) was colocalized with a small fraction of endogenous tubulins, TUBA1B and TUBB3 (4%–8%), in the cytoplasm of NPC cells.

To elucidate the interaction domains of CLDN11 for tubulins, various deletion constructs of CLDN11–3F were generated (Fig. 5d). These deletion clones were overexpressed and immunoprecipitated by using Flag M2 beads. We observed that either the intracellular loop or the cytoplasmic C-terminus could contribute to pull down the two endogenous tubulins, suggesting that they are the two crucial domains of CLDN11 interacting with TUBA1B and TUBB3 (Fig. 5d). Subsequently, these deletion clones were introduced into NPC cells to confirm whether the intracellular loop and cytoplasmic C-terminus of CLDN11 are required for CLDN11-mediated migration suppression. Notably, in the absence of the tubulin interacting domains (deletion clone no. 4 and 5), CLDN11 lost its ability to block cell migration (Fig. 5e), indicating that these domains are essential for CLDN11 to execute its anti-migration function.

α- and β-tubulins undergo dynamic polymerization forming microtubules, of which are required for mitosis, cell movement, intracellular transport, and structural support. We then investigated whether the presence of CLDN11 affects in vitro tubulins polymerization. By determining the optical density at 340 nm (OD340) of the reaction, we observed that tubulins rapidly polymerized in the standard reaction. The reaction reached a plateau after 20 min (Additional file 9: Figure S5a) and the Vmax of the reaction was 44.2 mOD/min at 11.5 min (Additional file 9: Figure S5b). The addition of a tubulin polymerization inhibitor, nocodazole (10 μM), substantially blocked the process; the polymerization reached plateau after 60 min (Additional file 9: Figure S5a) and the Vmax was 5.6 mOD/min at 40 min (Additional file 9: Figure S5b). The presence of GST-CLDN11 affected the tubulin polymerization process. The time required to reach plateau was 40 min for GST-CLDN11 (Additional file 9: Figure S5a), and the Vmax was 16.8 mOD/min at 24 min for GST-CLDN11 (Additional file 9: Figure S5b). These results strongly suggested that CLDN11 interferes with tubulin polymerization, that the intracellular loop and C-terminus of CLDN11 are necessary for the binding of TUBA1B and TUBB3, and that the deletion of intracellular loop and C-terminus of exogenous CLDN11 impairs the CLDN11-mediated migration blockage in NPC cells. By interacting with microtubules, CLDN11 essentially maintains and stabilizes normal epithelial cell structure. Thus, the silencing of *CLDN11* promotes the migration ability of NPC cells.

To determine whether a tubulin polymerization inhibitor can be used to block the migration ability of NPC cells, we performed migration assays in the presence of nocodazole. This treatment effectively blocked the migration ability of TW02 and HK1 cells in a dose-dependent manner (Fig. 5f) without affecting cell viability (Fig. 5g), thus indicating that the nocodazole treatment results were similar to those of CLDN11 re-expression in NPC cells. Hence, tubulin polymerization inhibitor(s) in general may compensate CLDN11 downregulation, serving as potential therapeutic drug(s) for NPC.

## Discussion

Genome-wide profiling for hypermethylated DNA sequences in tumor relative to nontumor samples facilitates identification of epigenetically misregulated cancer-related genes [29, 30]. Moreover, hypermethylated genes may serve as diagnostic or prognostic biomarkers for early detection as well as potential therapeutic targets for cancer treatment [31, 32]. Here, we found that the tight junction *CLDN11* is a novel differentially hypermethylated and downregulated gene in NPC tumors. Our bisulfite sequencing analysis demonstrated that the hypermethylated *CLDN11* promoter region is located at − 137 to + 405, similar to previously reported differentially hypermethylated region in gastric cancer (− 104 to + 4) [23] and in melanoma (+ 144 to + 249) [33]. This indicates that aberrant hypermethylation of *CLDN11* promoter frequently occurs near the transcription start site in different cancers. Based on the promoter assay results, GATA1 and GATA2 are the two crucial transcription activators responsible for *CLDN11* activation. A study demonstrated that GATA family proteins upregulate murine *CLDN11* promoter activity [34], indicating the GATA-mediated *CLDN11* activation is conserved among species. Although no apparent CpG site has been noted within the consensus sequences of GATA1 (G/CNNGATTNNNN) and GATA2 (NNNGATA/GNNN) on the *CLDN11* promoter, two neighboring CpG sites upstream of the GATA1/2 (− 62) core consensus have been revealed. The methylation of these flanking CpG sites may still impede the binding of GATA, causing transcriptional silencing of *CLDN11*.

*CLDN11* is mainly expressed in the brain and testes, where it form tight junction strands in oligodendrocytes and Sertoli cells, respectively [35, 36]. *CLDN11*-null mice exhibit defects in the neurological and reproductive systems, suggesting the pivotal role of *CLDN11* in establishing the paracellular physical barrier of tight junctions necessary for normal CNS function and spermatogenesis [37–39]. In addition to cell barrier functions, *CLDN11* appears to have a tumor suppressor function. First, *CLDN11* is often inactivated in tumors; nevertheless, its re-expression decreases cell motility and invasiveness in gastric and bladder cancers [18, 23], similar to the observations in this study. Normal nasopharynx epithelial cells are uniformly arranged, with 3–4 cell layers attached to the basement membrane. By contrast, NPC cells lose orderly morphology, cell polarity, and the basement membrane, but acquire aberrant cell expansion ability. Our immunohistochemistry data demonstrated strong staining signal for CLDN11 at the apical surface of the normal nasopharynx cells and exhibited typical tight junction staining pattern. However, CLDN11 expression was low and diffusely located in the cytoplasm of NPC tumor tissues. Notably, the forced expression of CLDN11 in NPC cells did not restore CLDN11 to its appropriate location, the plasma membrane. This mislocalized exogenous CLDN11 occurred potentially because of the downregulation of other tight junction components or defective protein trafficking mechanism in NPC cells.

Apart from being considered tight junction scaffolds, claudins may have multiple roles beyond the cell barrier including governing cell cycle, motility, transformation, and proliferation [17, 40–43]. Furthermore, different claudins may have a positive or negative influence on tumorigenesis; even the same claudin may behave differently in different cancers [44, 45]. Hence, claudins may interact with various cellular partners to execute its diverse functions. Therefore, protein–protein interaction networks determine the potential functions of claudins. In general, claudins comprise four transmembrane domains, two extracellular loops, one intracellular loop, and a short intracellular C-terminus with PDZ motif, postsynaptic density protein PSD95, Drosophila disk large tumor suppressor (Dlg1), and zonula occludens-1 protein (ZO-1) [14]. A classical claudin’s PDZ motif can interact with ZOs through the last two amino acids YV at the C-terminus [46, 47]. However, unlike classical claudins, CLDN11 terminates with HV, a nonclassical PDZ domain, and it does not interact with ZOs [48]. Interestingly, the exogenous overexpression of CLDN11 reduced the migration and invasion abilities of NPC cells. Thus, in addition to its traditional roles in cell barrier, cell polarity, and paracellular transport, CLDN11 may have other cellular functions involved in cell motility regulation.

This is the first report demonstrating that microtubule components TUBA1B and TUBB3 are the two novel CLDN11 interacting proteins. Microtubules, composed of α-β tubulin heterodimers, are cytoskeleton elements involved in multiple cellular processes including mitosis, transportation, signal transduction, and motility [49, 50]. Our tubulin polymerization assay results demonstrated that CLDN11 may perturb the dynamic α-β tubulin heterodimerization. We speculate that the interaction between CLDN11 and the two tubulins may modulate microtubules remodeling, possibly influencing the cytoskeleton rearrangement, directional migratory effect, cell motility, and cell cycle in cancer cells. Furthermore, CLDN11 may provide an anchorage platform to secure the cytoskeleton microtubules and make them less flexible, thus maintaining the polarity and orderly morphology of normal epithelial cells.

**Fig. 6:**
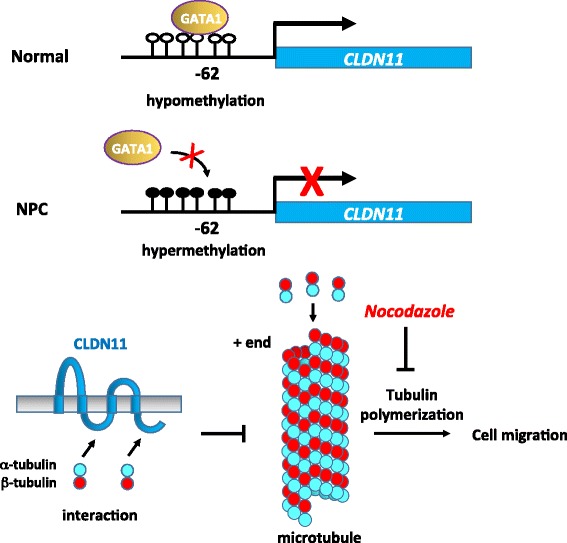
A Model for transcriptional silencing of *CLDN11* through hypermethylation promotes migration by derepression of tubulin polymerization. In a normal nasopharynx, CLDN11 is transcriptionally activated by transcription activators, GATA1 and GATA2. The integral membrane tight junction protein CLDN11, expressed on the apical surface of the epithelial cells, maintains tight junction integrity and epithelial cell polarity and morphology. In addition, CLDN11 serves as the scaffold to recruit tubulins through its intracellular loop and C-terminal domains. The interaction between CLDN11 and the tubulins TUBA1B and TUBB3 may sequester the availability of α- and β-tubulin subunits in the cytoplasm. Thus, the presence of CLDN11 may prevent cell migration and invasion by interfering with the microtubule polymerization dynamics. By contrast, in NPC cells, aberrant promoter hypermethylation impairs GATA binding and causes transcriptional silencing of *CLDN11*. In the absence of CLDN11, microtubules undergo rapid polymerization, in turn promoting basement membrane breakdown, motility, invasiveness, plasticity, and cell cycle, thus contributing to a more cancerous phenotype of NPC cells. The tubulin polymerization inhibitor nocodazole can serve as a therapeutic drug to block migration in NPC

Finally, we propose a working model of *CLDN11* transcriptional regulation and functions in Fig. 6. Thus, *CLDN11* is a bona fide tumor suppressor gene, which can be inactivated by DNA methylation. Our study provides a novel molecular mechanism underlying the anticancer function of CLDN11. Tubulin polymerization inhibitors may be used to block migration in cancer cells, which have low CLDN11 expression. Thus, CLDN11 may serve as prognostic and potential therapeutic biomarker for NPC and other cancers.

## Conclusions

In this study, we identified a differentially hypermethylated and downregulated gene *CLDN11* in NPC. CLDN11 exerts its migration inhibitory role through interacting with TUBA1B and TUBB3, and disturbing the tubulin polymerization. Therefore, silencing of *CLDN11* increases cell plasticity and promotes NPC progression.

## Additional files

Additional file 1: Supplementary Materials and Methods. (DOC 39 kb)

Additional file 2: **Table S1.** Sequence of primers in this study. (PDF 105 kb)

Additional file 3: **Figure S1.** Differentially methylated CLDN11. Based on the methylated DNA fragment purified by methyl-binding protein affinity column, followed by next generation sequencing and DNAnexus (CA, USA) sequence analysis, *CLDN11* gene region (- 723~ + 1822) is enriched and differentially methylated (experimental versus background =1.5, C666.1 versus NP69). Schematic map of *CLDN11* (- 723~ + 1822) with CpG islands (blue region) is adapted from the MethPrimer website (http://www.urogene.org/methprimer/). CpG sites (orange vertical bars) and the transcription start site (+?1) are indicated. Read coverage of the methylated DNA from C666.1 and NP69 (green peaks) are visualized by DNAnexus genome browser. The orange region represents the differentially methylated peak in C666.1. (PDF 306 kb)

Additional file 4: **Figure S2.** Immunohistochemistry staining analysis of CLDN11 in nine paired NPC tissues with higher magnification (800X). (TIFF 8900 kb)

Additional file 5: **Figure S3.** Sequence of *CLDN11* promoter (- 1000~ + 200). The promoter sequence of *CLDN11*, transcription factor binding sites [GATA1(- 90), GATA1/2(- 60), GATA2(+ 184)] and transcription start sites (+ 1) are indicated. (PDF 44 kb)

Additional file 6: **Figure S4.** Co-immunoprecipitation of interacting proteins of Flag-tagged CLDN11. (a) Co-immunoprecipitation assays were performed using anti-Flag M2 beads (Sigma) on cell lysates transfected with either vector (Vec, V) or CLDN11-3F (C) expressing plasmid. The immunoprecipitated protein samples were separated in 12% SDS-PAGE. The gel was stained with Coomassie Blue (left panel). (b) For Western blot analysis, the SDS-PAGE was loaded with 1% input lysate, 25% immunoprecipitated lysate, and 1% flow through (FT), and was detected by anti-Flag antibody. (PDF 165 kb)

Additional file 7: **Table S2.** Identified proteins from CLDN11-3flag Co-IP by LC-MS/MS. (PDF 222 kb)

Additional file 8: Identified peptide sequence of TUBA1b, TUBB3 and CLDN11-3flag by LC-MS/MS. (XLSX 19 kb)

Additional file 9: **Figure S5.** Tubulin polymerization assays. (a) Tubulin polymerization assays were performed in standard condition (TUB), TUB with purified GST, TUB with purified GST-CLDN11, and TUB with 10 µM nocodazole (tubulin polymerization inhibitor). Tubulin polymerization was monitored using an ELISA reader (340nm) every 30 s for 60 min (upper panel). (b) The rate of tubulin polymerization (?mOD/min) was plotted (lower panel) and the Vmax for each reaction is indicated by an arrow. (c) The purified GST and GST-CLDN11 protein were detected by western blotting using either anti-GST or anti-CLDN11 antibody. (PDF 203 kb)

## Abbreviations

5’Aza: 5′-azacytidine
ANOVA: Analysis of variance
BS: Bisulfite sequencing
ChIP: Chromatin immunoprecipitation
CLDN: Claudin
DMEM: Dulbecco’s Modified Eagle’s medium
DNMT1: DNA methyltransferase 1
EBV: Epstein–Barr virus
EMSA: Electrophoretic mobility shift assay
FBS: Fetal bovine serum
HRP: Horseradish peroxidase
LC-MS/MS: Liquid chromatography-tandem mass spectrometry
LMP1: Latent membrane protein 1
MAP: Microtubule associated protein
MBD: Methyl binding domain
NGS: Next generation sequencing
NPC: Nasopharyngeal carcinoma
OD: Optical density
PBS: Phosphate buffered saline
qRT-PCR: Quantitative real time polymerase chain reaction
RPMI: Roswell Park Memorial Institute
RT: Reverse transcription
SD: Standard deviation
TUBA1B: Tubulin alpha-1b
TUBB3: Tubulin beta-3
