# Supplementary material for: Inactivation of the tight junction gene CLDN11 by aberrant hypermethylation modulates tubulins polymerization and promotes cell migration in nasopharyngeal carcinoma
Source: J Exp Clin Cancer Res. 2018 May 10;37:102. doi: 10.1186/s13046-018-0754-y (PMC5946489; doi:10.1186/s13046-018-0754-y)
Supplement: Supplementary file 1 — Supplementary Materials and Methods. (DOC 39 kb) [file 13046_2018_754_MOESM1_ESM.doc]

**Inactivation of the Tight Junction Gene *CLDN11* by Aberrant Hypermethylation Modulates Tubulins Polymerization and Promotes Cell Migration in Nasopharyngeal Carcinoma**

**Additional file 1: supplementary materials and methods**

**Materials and Methods**

***Next Generation Sequencing of enriched methylated DNA***

Genomic DNA from C666.1 and NP69 cells were isolated and sonicated (200~600bp, total 24μg). Methylated DNA was enriched using MethylMiner™ Methylated DNA Enrichment Kit (Thermo Fisher/Invitrogen) as manufacturer’s description. Briefly, methylated DNA was bound by biotinylated methyl binding protein, MBD2, captured by the streptavidin-coated Dynabeads, and eluted by stepwise gradient elution buffer containing sodium chloride (600mM, 1000mM and 2000mM). The eluted DNA was further precipitated (~1μg) and sequenced by Yang Ming University VYM Genome Research Center (next generation sequencing, Illumina GAII, ChIP single read, 75bp). NGS data was analyzed by cloud-based platform DNAnexus (San Francisco, CA) and CGU MMRC, Bioinformatics Core Lab., and the data was mapped to human reference sequence hg18. Sequenced DNA reads were denoted as relative methylated DNA enrichment.

***Co-immunoprecipitation assay and liquid chromatography-tandem mass spectrometry, LC-MS/MS***

TW02 cells transiently expressed vector or CLDN11-3F were extracted by lysis buffer (150mM NaCl, 20mM Tris-HCl pH7.5, 0.25% DOC, 0.5% Triton X-100, 0.5% NP40, 0.05% SDS). Cell lysates (12mg) was immunoprecipitated by 150μl Flag M2 Affinity Gel (Sigma) and incubated for 4hr at 4°C, and then washed ten times. The M2 beads were eluted with 2X SDS sample buffer and the immunoprecipitated protein samples were separated in 12% SDS-PAGE (Supplemental Figure S2). The gel was stained with Coomassie Blue, sliced 20 pieces and in-gel trypsin digested according to protocol established by CGU MMRC, Proteomics Core Lab. (<http://163.25.92.61/core/Proteomics/download.htm>). Eluted peptides were subjected to LC-MS/MS coupled with ion trap LTQ-Orbitrap MS (Thermo Fisher) operated by Xcalibur 2.0.7 software (Thermo Fisher), MS-full scan and 10 MS/MS scans as described . The MS/MS spectra were searched using the Mascot algorithm (version 2.2.06, Matrix Science, U.K.) and the Swiss-Prot database (Homo sapiens, 2010). Protein identification was analyzed by Scaffold proteome software (version 3.3.2, Proteome Software Inc., Portland, OR), in which peptide and protein threshold cut offs were at a minimum of 95% with a minimum of two peptides.

***Tubulin polymerization assay***

Tubulin polymerization assay (Cytoskeleton^TM^, BK006P, Denver, CO) was performed as instructed. Purified tubulin (300μg) in 0.1 ml polymerization buffer (80mM PIPES pH6.9, 2mM MgCl_2_, 0.5mM EGTA, 1mM GTP, 10% glycerol) was used in each reaction. Recombinant proteins 0.5μg GST and GST-CLDN11, and tubulin destabilizing drug nocodazole 10μM were added separately into the standard tubulin in 96-well plate. The optical density of polymerized tubulin was recorded by ELISA reader (Molecular Devices, Sunnyvale, CA) with program settings: kinetics, 121 cycles, 1 reading/30sec, OD340nm, 37^0^C.
