# Supplementary material for: Inactivation of the tight junction gene CLDN11 by aberrant hypermethylation modulates tubulins polymerization and promotes cell migration in nasopharyngeal carcinoma
Source: J Exp Clin Cancer Res. 2018 May 10;37:102. doi: 10.1186/s13046-018-0754-y (PMC5946489; doi:10.1186/s13046-018-0754-y)
Supplement: Supplementary file 2 — Table S1. Sequence of primers in this study. (PDF 105 kb) [file 13046_2018_754_MOESM2_ESM.pdf]

Table S1. Sequence of primers in this study

Primer		Sequence (Forward and Reverse)
Bisulfite sequencing-CLDN11	F	TGGGGTTATTTTGTTTTTTTT
	R	AAAAACTTACAATAATACAACCCC
QRT-PCR-CLDN11	F	TGGTGTTTTGCTCATTCTGC
	R	CATACAGGGAGTAGCCAAAG
cDNA-CLDN11	F	GGATCCATGGTGGCCACGTGCCTGCAG
	R	AAGCTTTACGTGGGCACTCTTCGCATGAGTC
CLDN11 Promoter deletion constructs:		
(-900)	F	TAGATCTGCACGATCTTGGCGCACTGCAA
(-477)	F	CAGATCTTAACTGTGTGTACTGAA
(-213)	F	TAGATCTGGACCTGGATGGAATTG
(-10)	F	GAGATCTCGCGCTGCCCAGCAGCG
(+197)	R	AAGCTTCCGCCAGGATGTCCCCGTGC
5’-Biotinylated Oligonucleotides:		
GATA1/2 WT	F	CTGCCGCCGATTGGTGCTCGC
	R	GCGAGCACCAATCGGCGGCAG
ME	F	CTGC^{m}CGC^{m}CGATTGGTGCTCGC
	R	GCGAGCACCAAT^{m}CGG^{m}CGGCAG
MT	F	CTGCCGAAGCTTGGTGCTCGC
	R	GCGAGCACCAAGCTTCGGCAG
