# Supplementary figures and images for: Inactivation of the tight junction gene CLDN11 by aberrant hypermethylation modulates tubulins polymerization and promotes cell migration in nasopharyngeal carcinoma
Source: J Exp Clin Cancer Res. 2018 May 10;37:102. doi: 10.1186/s13046-018-0754-y (PMC5946489; doi:10.1186/s13046-018-0754-y)

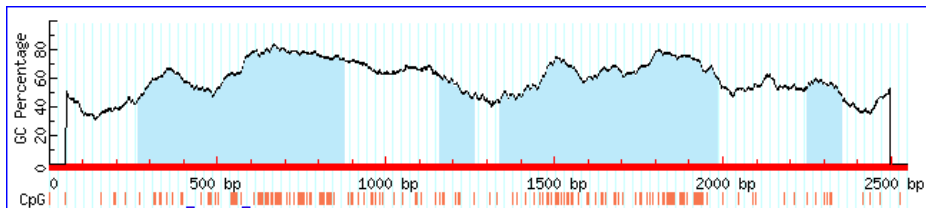

-723

+1

+1822

CLDN11

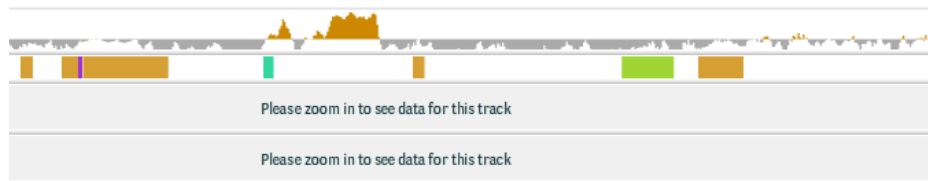

C666.1

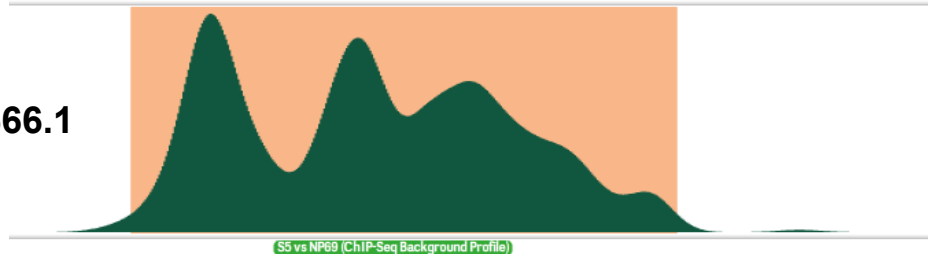

NP69

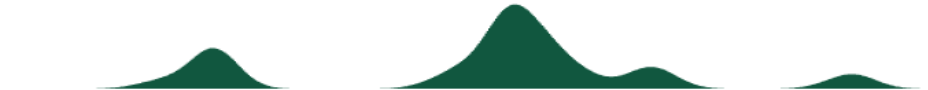

Supplement: Supplementary file 3 — Figure S1. Differentially methylated CLDN11. Based on the methylated DNA fragment purified by methyl-binding protein affinity column, followed by next generation sequencing and DNAnexus (CA, USA) sequence analysis, CLDN11 gene region (- 723~ + 1822) is enriched and differentially methylated (experimental versus background =1.5, C666.1 versus NP69). Schematic map of CLDN11 (- 723~ + 1822) with CpG islands (blue region) is adapted from the MethPrimer website (http://www.urogene.org/methprimer/). CpG sites (orange vertical bars) and the transcription start site (+?1) are indicated. Read coverage of the methylated DNA from C666.1 and NP69 (green peaks) are visualized by DNAnexus genome browser. The orange region represents the differentially methylated peak in C666.1. (PDF 306 kb) [file 13046_2018_754_MOESM3_ESM.pdf]

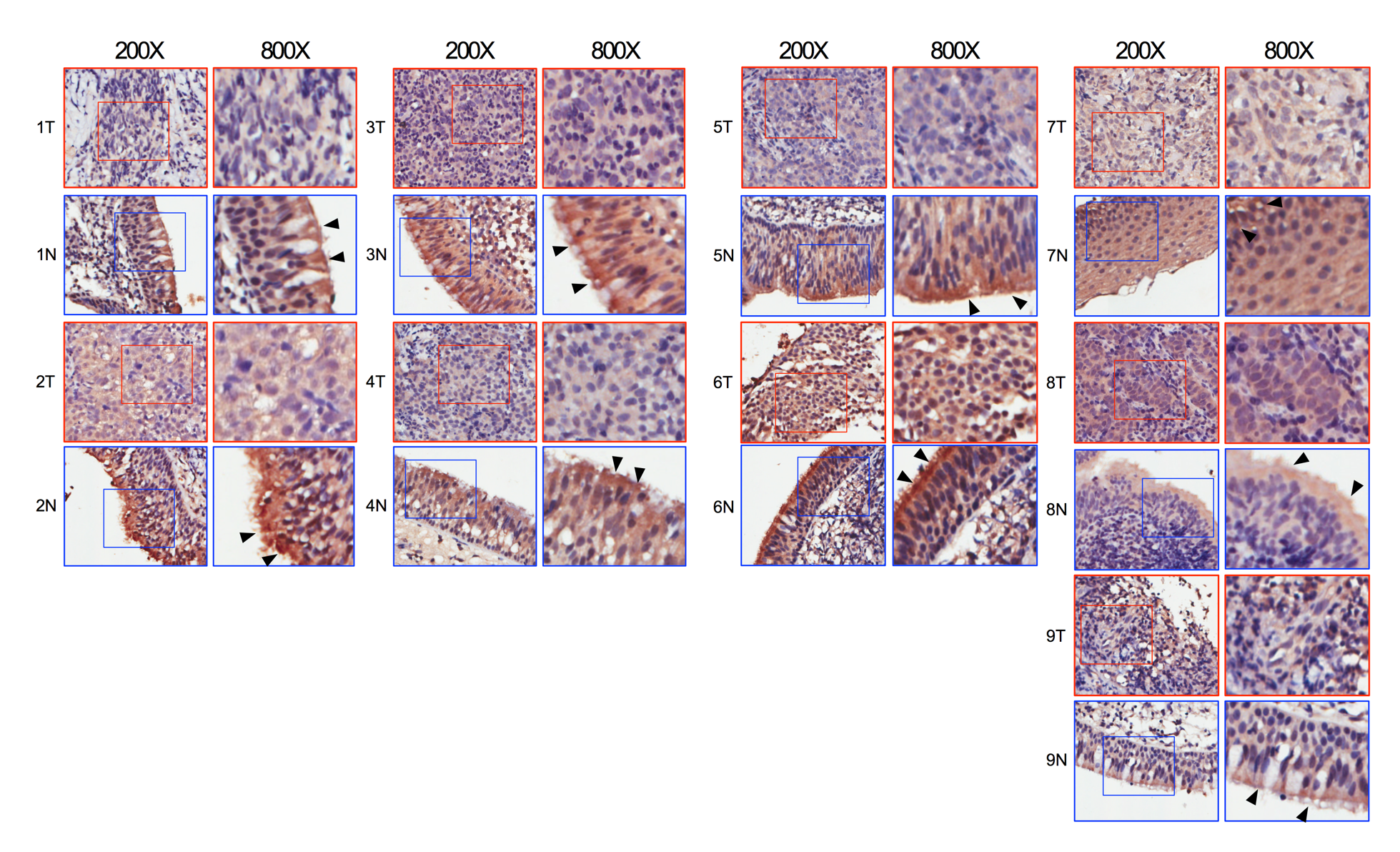

Supplement: Supplementary file 4 — Figure S2. Immunohistochemistry staining analysis of CLDN11 in nine paired NPC tissues with higher magnification (800X). (TIFF 8900 kb) [file 13046_2018_754_MOESM4_ESM.tiff]

A

TW02 (IP)

Vec

CLDN11-3F

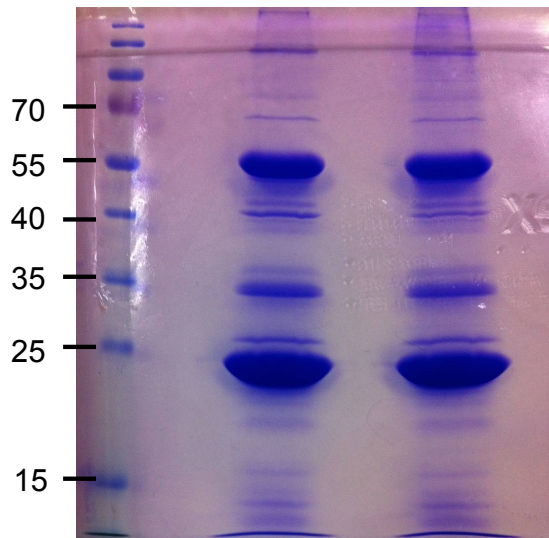

B

| 1% Input |   | 25% IP |   | 1% FT |   |
|----------|---|--------|---|-------|---|
| V        | C | V      | C | V     | C |

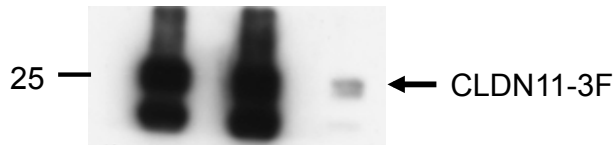

Supplement: Supplementary file 6 — Figure S4. Co-immunoprecipitation of interacting proteins of Flag-tagged CLDN11. (a) Co-immunoprecipitation assays were performed using anti-Flag M2 beads (Sigma) on cell lysates transfected with either vector (Vec, V) or CLDN11-3F (C) expressing plasmid. The immunoprecipitated protein samples were separated in 12% SDS-PAGE. The gel was stained with Coomassie Blue (left panel). (b) For Western blot analysis, the SDS-PAGE was loaded with 1% input lysate, 25% immunoprecipitated lysate, and 1% flow through (FT), and was detected by anti-Flag antibody. (PDF 165 kb) [file 13046_2018_754_MOESM6_ESM.pdf]

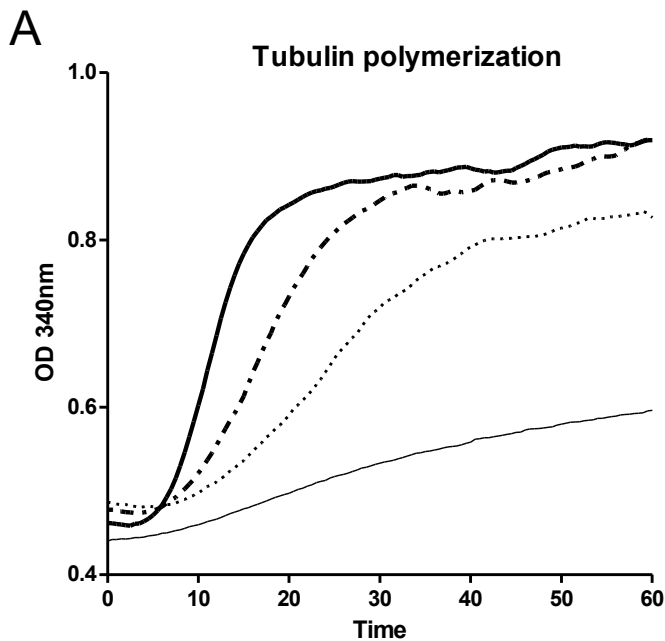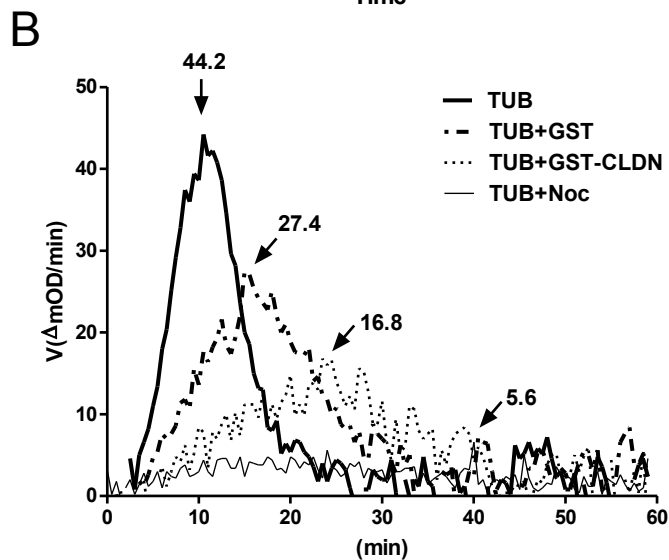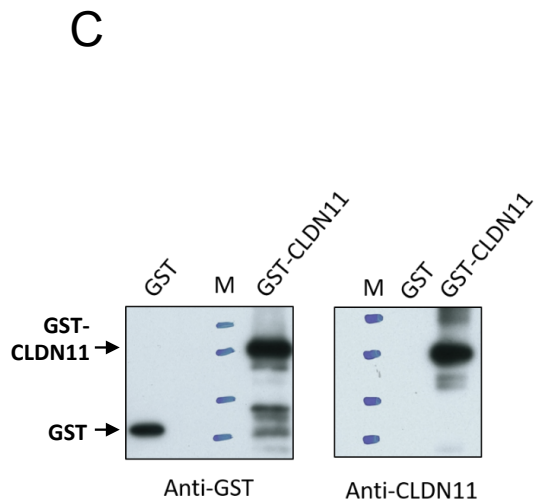

Supplement: Supplementary file 9 — Figure S5. Tubulin polymerization assays. (a) Tubulin polymerization assays were performed in standard condition (TUB), TUB with purified GST, TUB with purified GST-CLDN11, and TUB with 10 µM nocodazole (tubulin polymerization inhibitor). Tubulin polymerization was monitored using an ELISA reader (340nm) every 30 s for 60 min (upper panel). (b) The rate of tubulin polymerization (?mOD/min) was plotted (lower panel) and the Vmax for each reaction is indicated by an arrow. (c) The purified GST and GST-CLDN11 protein were detected by western blotting using either anti-GST or anti-CLDN11 antibody. (PDF 203 kb) [file 13046_2018_754_MOESM9_ESM.pdf]
