# Supplementary material for: Inactivation of the tight junction gene CLDN11 by aberrant hypermethylation modulates tubulins polymerization and promotes cell migration in nasopharyngeal carcinoma
Source: J Exp Clin Cancer Res. 2018 May 10;37:102. doi: 10.1186/s13046-018-0754-y (PMC5946489; doi:10.1186/s13046-018-0754-y)
Supplement: Supplementary file 5 — Figure S3. Sequence of CLDN11 promoter (- 1000~ + 200). The promoter sequence of CLDN11, transcription factor binding sites [GATA1(- 90), GATA1/2(- 60), GATA2(+ 184)] and transcription start sites (+ 1) are indicated. (PDF 44 kb) [file 13046_2018_754_MOESM5_ESM.pdf]

***CLDN11* promoter sequence (-1000~+200)**

tgtgctgtttctctatggctagtgcgtgtaatttgttgttgttgttgttg

tttgcttttgagacgttgtctcactcagttgcccaggctggagtgcagtg

gcacgatcttggcgcactgcaacctccttcaagttattctcctgcctcag

cctcccaagcagctggggttacaggcatgcgccaccatgcccggctcatt

tttgtattttctgtagagatggagtttcaccatgttggtcaggctggtct

cgaactcctgaccttaggtgatccgcccaccttggcctcccaaagttctg

ggattacaggtgtgagccaccactcccggcctagtatgtgtcattgacaa

ttgttcctacctcaaaggctgttatgaggattaaatgagctaatgcatgt

aaaatgcatctgcataatatataacacagctcgataaatgtgagttgcta

ttgttgattattatggccacgtttgccgctgatgatgttcaaccactgaa

aagcatcgttttctttcttctagtaactgtgtgtactgaatgcctcgcgt

ttgtccctactgcacttttcagtctgtcacaatcctgggtcgtcgggga

ctgtaccgagcgcccttggagtgctcgcagcagggaagaaggagcgagca

gggaggagctgaggagggtcggagcggaatggacacccagagactgagat

gagagaggggctataagaagagaaaccagtcgcaggaaaacagaactctc

gacaaacgcacgccctctttgcgctctccgtttctctggacctggatgga

attgttttactttgcatcgccggactgcgctgcggacggggtggggcggc

ccaatggccccctggggttactctgctccccttcacgcttcctcccgg

GATA1 GATA1/2

ccccgccccgcgattggccggcgcgccccggggctgccgccgattggtgc

TATA

tcgccggccggagccgcggggcttaaagagggggcgggggcgcgctgccc

+1 AGCAGCGCTGCTGTCCCCGCCGTGCGCCCTTCGCCGCTGAGCTCGCAGCC

TCCGGCGCCCACCTCCACCTCCAGTGTCCCGCCTCGGGCCGTCGCCCTCC

AGCGGCTCGCGAGCGTGGGAGACGTACCTGGGCAGGCACTGTCCAGCCCA

GATA2

GGCCCAGGCACAGCCGTGAGGGGCGAGGCACGGGGACATCCTGGCGGCCA
