# Supplementary material for: Inactivation of the tight junction gene CLDN11 by aberrant hypermethylation modulates tubulins polymerization and promotes cell migration in nasopharyngeal carcinoma
Source: J Exp Clin Cancer Res. 2018 May 10;37:102. doi: 10.1186/s13046-018-0754-y (PMC5946489; doi:10.1186/s13046-018-0754-y)
Supplement: Supplementary file 7 — Table S2. Identified proteins from CLDN11-3flag Co-IP by LC-MS/MS. (PDF 222 kb) [file 13046_2018_754_MOESM7_ESM.pdf]

Table S2. Identified protein with fold-change >30 and p-value < 0.01

Accession No.	Gene Symbol	MW	Identified peptides	Sequence coverage (%)	total spectra fold-change (C/V)	p-value
TBA1B_HUMAN	TUBA1B	50 kDa	25	69.2	800.06	<0.001
CLD11_HUMAN	CLDN11	22 kDa	4	15.9	193.99	0.005
MBB1A_HUMAN	MYBBP1A	149 kDa	35	31	191.82	0.002
ESYT2_HUMAN	ESYT2	102 kDa	15	22.3	77.92	0.001
TRRAP_HUMAN	TRRAP	438 kDa	32	10.3	70.51	0.004
NU188_HUMAN	NUP188	196 kDa	18	13.3	67.20	0.001
PDS5B_HUMAN	PDS5B	165 kDa	20	18.3	47.33	0.004
ITM2B_HUMAN	ITM2B	30 kDa	10	51.9	46.42	0.005
MRP1_HUMAN	ABCC1	172 kDa	15	13.5	43.45	0.008
TBB3_HUMAN	TUBB3	50 kDa	6	49.8	36.96	0.005
LY6K_HUMAN	LY6K	19 kDa	4	31.5	32.61	0.002
AT2B1_HUMAN	ATP2B1	139 kDa	13	13.8	32.50	0.003
S38A2_HUMAN	SLC38A2	56 kDa	5	17.6	31.98	0.003
MON2_HUMAN	MON2	190 kDa	11	7.1	31.95	0.009
DECR2_HUMAN	DECR2	31 kDa	10	33.9	31.86	0.007
TAP2_HUMAN	TAP2	76 kDa	9	15.5	30.85	0.008
GAPD1_HUMAN	GAPVD1	165 kDa	10	9.4	30.48	<0.001
RM13_HUMAN	MRPL13	21 kDa	6	39.3	30.12	0.010

MW, molecular weight
